# Work shadowing in dental teaching practices: evaluation results of a collaborative study between university and general dental practices

**DOI:** 10.1186/s12909-018-1220-4

**Published:** 2018-05-08

**Authors:** Stefan J. Heitkamp, Stefan Rüttermann, Susanne Gerhardt-Szép

**Affiliations:** Department of Operative Dentistry, Dental School (Carolinum), Goethe-University Frankfurt, Theodor-Stern-Kai 7/29, D-60596 Frankfurt am Main, Germany

**Keywords:** Dental training, Work shadowing, Medical traineeship, General dental practice, Evaluation, Multi-modal feedback, Medical communication, Benefit

## Abstract

**Background:**

The aim of this study was to investigate the acceptance and assessment of work shadowing carried out by students and dentists in dental practices. Furthermore, the extent to which students perceive an improvement in their specialised, communication and social competencies, was to be examined.

**Methods:**

61 dental students in their clinical semesters at a German university participated in work shadowing placements at 27 different general dental practices. Before beginning, they received checklists of various competencies that they self-assessed using school grades (from 1 = ‘very good’, to 6 = ‘failed’), which they also repeated after completion. The dentists supplemented this with their external assessments. In addition, the students were requested to fill out a 54-item questionnaire and compose a freely-structured report after the work shadowing; the dentists filled out a questionnaire containing 16 items. The statistical analysis was carried out by means of the Friedman Test, including a post-hoc test (Bonferroni-Holm correction).

**Results:**

The analysis showed a significant overall improvement in the students’ self-assessed competencies by 0.71* ± 0.43 grades. With an average of 0.33* ± 0.36, the dentists’ external assessment proved significantly higher than the self-assessment. The greatest improvements were perceived by the students in the areas of accounting (1.17* ± 0.77), practice organisation (1.05* ± 0.61) and dentist’s discussions (0.94* ±0.80) [*p < 0.05]. The students confirmed experiencing an expansion of knowledge, an improvement in their communication skills and indicated a high degree of satisfaction in regard to the dentists (school grade 1.58 ± 0.93). A maximum amount of satisfaction towards the work shadow students was demonstrated by the dentists, and this form of teaching was assessed with a school grade of 1.69 ± 0.89.

**Conclusion:**

Both students and dental practitioners demonstrated a high level of satisfaction in regard to the work shadowing. The students felt their knowledge had increased, viewed the dentists as motivating role models and acknowledged a significant improvement in their specialised, communication and social competencies. Work shadowing in dental teaching practices presents a sensible addition to academic teaching at a university.

## Background

The practical life of a dentist has changed over the last decades and many areas are not reflected in current German education. The ‘Dental Licensure Act’ (‘Zahnärztliche Approbationsordnung’) has been virtually unchanged since its creation in 1955 [1]. But in more than 60 years, many scientific findings, as well as social, technical and educational developments have changed the requirements for a modern study of dentistry. It is not surprising that in Germany the call for a new version of the Licensure Act has become increasingly louder. For the first time, the goal now seems very close: The so called ‘Ordinance for the new regulation of the study of dentistry‘ (‘Verordnung zur Neuregelung der zahnärztlichen Ausbildung’) from the German Federal Ministry of Health was accepted by the Federal Cabinet on 2/8/2017 and has paved the way for a basic reformation of the Dental Licensure Act. This means that it can be passed on to the Federal Council and after approval there, the Ordinance could be implemented on 1/10/2018 [2].

Among numerous amendments and restructurings, a four-week clinical traineeship in form of work shadowing (‘Hospitation’) in dental practices and treatment institutes is mentioned in the Ordinance, in order to be more closely aligned with the study of medicine and to reinforce the focus of practice occurring at an early stage of dental studies, which is a novelty in the training of dentists in Germany [3].

The need to expand the practice-related portion of training, by incorporating practicing dentists into the course was already determined in 2005 by the German Council of Science and Humanities (‘Wissenschaftsrat’) with its ‘Recommendations for the further development of dentistry at German universities’. It regards this as a counterbalance to the predominant supervision of the students by young assistant dentists at universities, and recommends incorporating teaching practices into the curriculum [4]. In consideration of the implementation of clinical traineeships planned in the new Dental Licensure Act, the urgent need to establish dental teaching practices has become apparent [3].

As literature shows, the workplace can be an important place for learning and development, in which valuable knowledge can be gained [5]. Workplace-based learning can enhance skills [6] and is often incorporated into social interactions, mentoring and everyday practices [7]. An experienced person is commonly described as the best source of information about a new job wherein he or she can inform about the challenges of and changes required for a specific task [8].

Especially in medical education workplace-based learning has proved its value to translate medical theory into clinical practice [9, 10]. Teaching and learning in a clinical environment provides real patients with real problems in the context of professional practice, which is valued by the students [11]. The learning is often acquired unawares through collaboration involving informal discussions about patient cases and modelling for others, which helps to modify attitudes and to develop a professional identity [12–16]. In dental education as well, it is known how important this role modelling is and how much influence it has on personal and professional growth [17].

In contrast to the German study of dentistry, this workplace-based learning has been incorporated in the study of medicine for long time through traineeships (‘Famulaturen’), to greatly benefit teaching [11, 18, 19]. In many areas, contact with patients outside the academic environment is sought in this way at an early stage of medical studies, in order to familiarise students with their later profession, to encourage the motivation of students and to improve social and communication skills [20–24]. Particularly in the area of primary care, it is strongly recommended to do work shadowing early with a practicing doctor as a teacher, in order to gain insights into the basics of examination methods, diagnostics, the interpretation of medical findings and the doctor-patient relationship [25, 26].

The teaching practice itself is also enriched by such teaching opportunities, mainly through attaining an academic feeling of belonging [27, 28] and the exchange of knowledge being perceived as an enrichment and improvement of the teaching from both sides [29–31].

In relation to the study of dentistry, one can find studies about teaching opportunities outside of the realm of a university, largely from Great Britain, but also from Scandinavia, the Netherlands, Australia and the USA [32, 33]. In the form of numerous so-called ‘outreach’ programmes, students are given the opportunity to gain supervised experience of treatment within the scope of external dental treatment institutes, very similar to their future professional life [33–43]. These outreach internships usually take place in centres of dental primary care such as community clinics or the National Health Service (NHS) that provide services for patients with special dental needs in otherwise under-served areas [43]. Research data shows that dental students highly appreciate these internships, and the benefits are an increased confidence, higher professionalism and improved competencies [33]. However, with regard to internships in dental practices, data from outreach studies can only be used to a limited extent. As Craddock 2011 showed, joining an established practice provides experiences beyond outreach programmes and the university [32]: A dental practice has specialisations with advanced equipment, and the patients are more demanding. It also offers additional areas such as practice organisation and accounting, as well as fitting into an existing team.

Up to now, no dental teaching opportunities outside the university were established nation-wide in Germany, due to a lack of curricular anchoring. The current German study of dentistry consists of ten semesters and a final exam. The first six semesters offer courses in basic medical sciences, as well as practical courses in dental technology and treatment courses on training dummies (called ‘phantoms’). From the 7th to the 10th semester, training takes place on real patients under supervision, and nearly every dental procedure is performed by the students [1]. Although these courses are very practice-oriented, the treatment in the teaching situation at the university differs from that at the workplace in a dental practice that most of the students will later have. Such an insight into reality is not provided for at any time during the current study curriculum. Likewise, it is seldom possible for students to watch an experienced dentist at work in order to deepen theoretical knowledge through practical demonstrations.

However, at several universities the potential for cooperation between the faculty of dentistry and general dental practices for the improvement of teaching has already been recognised and some work shadowing of students at practices has been carried out successfully [44, 45]. Furthermore, the German Dental Association (Bundeszahnärztekammer, BZÄK) strives to develop framework conditions for universities and medical chambers that also allow the establishment and incorporation of more academic teaching practices at other universities [45–47].

Particularly in regard to the German ‘National competence-based learning goal catalogue for dentistry’ (NKLZ) [48] developed in 2015, questions have arisen concerning the extent to which the future expansion of training in practical environments can lead to an expanded covering of the competencies outlined in the NKLZ. Especially, the role modelling of the communicator, collaborator and manager (Z7, Z8, Z10), as well as the function of the dentist as a scholar (Z6), might be realised through such an expansion.

In summary it can be said that teaching in medical practices has been well studied, but in the area of dentistry, there are primarily studies about outreach internships that, as mentioned, provide a different environment. Work shadowing in dental practices – as planned in the new Dental Licensure Act – has rarely been studied. Whether students can find role models in this special setting has not been explored, and especially the detailed influences on different competencies of the dental students remain unclear.

Given this background and based on a preceding pilot study [49], the following aims were established for the present study: To investigate the acceptance and assessment of work shadowing in dental practices, both on the part of the students and the practicing dentists, using a large collective. Furthermore, the extent to which students perceived an improvement in their specialised, communication and social competencies was to be examined, in order to determine what students and the courses gain from such time in a dental practice. In addition, it was to be investigated, whether the work shadowing allows an adequate initial view of the procedures and organisations of practices to improve competencies in new, practice-specific topics.

## Methods

### Concept

The study was developed in the Department of Operative Dentistry of the Dental School (Carolinum) [‘Poliklinik für Zahnerhaltung des Zentrums der Zahn-, Mund- und Kieferheilkunde (Carolinum)’] at Goethe-University Frankfurt-on-Main [49].

Educational interventions outside the university were planned for students in the early stages of their clinical studies, namely at dental teaching practices in the surrounding area. Based on the concept of Isler et al. [21], the interventions were referred to as ‘One-on-one tutorials’. These tutorials, unlike an internship in which a student actively treats patients under supervision, primarily intended an observational role for the students in form of work shadowing: The practices were supposed to take one student per week, who was allowed to accompany the dentist through the everyday life of the practice.

At the beginning of the tutorial, the student was to be given a tour of the practice and induction in the working environment by the practice staff. As a framework for the schedule and the content to be treated, checklists and information sheets were used, which were viewed and discussed with the dentist on the first day. These checklists were also used for data collection (EVA I, see 2.5.1).

The tutorials were divided into three parts with flexible time scheduling:

1. The main part one included the observation of treatment measures and the dentist’s patient communication, such as taking medical history and patient enlightenment. The student was instructed to keep in the background and was allowed to take notes while looking over the dentist’s shoulder.

2. In part two, informational and field-related discussions with the dentist were to be conducted in private. Here, the student could address all the questions and ambiguities that had arisen during the observation. In this context, the student was to present and discuss a self-written case report.

3. Part three provided that the dentist or practice manager offered the student an insight into the processes and organisation of a dental practice, as well as quality management and working in a team. Special attention was to be given to accounting, as this economic aspect is not covered in university teaching. Further important points were the cooperation with a dental laboratory and the emergency management of the practice, including discussion of the emergency suitcase.

In a final meeting on the last day, student and dentist were to discuss and evaluate their experiences (see 2.5 Data collection).

### Recruitment of dental practices

For the recruitment of dental practices, registered dentists around the area of Frankfurt were contacted and invited to an informational evening with a corresponding flyer. In the course of this two-hour event, the practice participants were informed of the study’s content, aims and schedule in detail. In subsequent open discussion there was the possibility to respond to questions of the dentists. And even after this event an information exchange was maintained by phone or e-mail via a project assistant.

An additional planned train-the-teacher event to prepare the dentists in detail for their teaching activity was not possible for financial reasons.

To participate, the following suitability criteria for the practices were established (inclusion criteria) [18]:

1. The general dental practice must encompass a broad spectrum (patients of all age groups, including care of the elderly and children; at least 35 operating hours of practice per week).

2. Students must have the opportunity to do some work or duties independently.

3. Time must be allotted for case-oriented discussions.

4. Access to literature (hand library) must be available, including Internet access, where possible.

The practices that fulfilled all of the above criteria and decided to participate, planned to take on work shadowers for up to five days in time slots convenient for them.

### Recruitment of dental students

For the recruitment of students, those participating in the ‘Phantom Course of Operative Dentistry’ (6th study semester: Students who have only done treatments with training dummies), as well as ‘Course I of Operative Dentistry’ (7th study semester: Students who have already done treatments with real patients for one semester), were provided detailed information on the study at introductory events of one hour. A total of 168 students were offered participation in the study on an optional basis during the lecture-free period, after completion of the first clinical semester/6th study semester (inclusion criteria). At the time of the realisation, there was no longer a teaching relationship between students and the authors. Neither did students receive any course credit, nor did the participation contribute to their formal assessment.

Since the evaluation was planned to be anonymous, the department’s ethics committee (‘Ethik-Kommission des Fachbereichs Medizin der Goethe-Universität Frankfurt am Main’) decided that a vote was not necessary and approved obtaining verbal consent from all participants.

### Realisation

During the time frame of February 2011 to March 2013, a total of *n* = 61 dental students and *n* = 27 general dental practices within the regional area of Frankfurt, took part in the study in three separate blocks.

The study population is shown in Table 1.

**Table 1:** Study population

	Men	Women	Total	Average age [years]
A	18	34	52	24.6 ± 3.78 (min. 21, max. 40)
B	1	8	9	25.9 ± 2.89 (min. 26, max. 30)
Total	19	42	61	24.8 ± 3.67 (min. 21, max. 40)

A: Students after completion of the first clinical semester (6th study semester), B: Students after completion of the second clinical semester (7th study semester)

After 2013 the funding by the medical faculty of the Goethe-University at Frankfurt-on-Main ceased and it was no longer possible to offer guided work shadowing placements. But up to now, the students use the framework created to do internships in the cooperating practices on their own initiative, while the project is still on hold, waiting to be continued.

### Data collection

The data collection was carried out by means of four separate evaluation instruments:

#### Checklists and assessment questionnaires related to competencies (EVA I)

At the start of the work shadowing, students received checklists for their own assessment of pre-defined core and supplementary competencies (see Table 4, left column) based on questionnaires of the Institute of General Medicine at the Goethe University, Frankfurt-on-Main [50, 51]. These were subdivided into sections for the student’s self-assessment before and after the period in the practice and an external assessment by the practicing dentist. On the first work shadowing day, the students made their own assessment, rating their listed competencies using school grades (1 = ‘very good’, to 6 = ‘failed’) and together with the dentist, they selected competencies that were meant to represent a main focus in the following work shadowing period. After completing the work shadowing, the students repeated their self-assessment and the dentist supplemented this with an external assessment from his/her point of view.

#### Student evaluation questionnaires (EVA II)

Following the work shadowing, the students received a questionnaire according to Isler et al. (see Tables 5, 6, 7, 8, 9 and 10, left column). This questionnaire was designed through trial evaluation and confirmatory factor analysis (CFA) for medicine and allows valid statements on knowledge acquisition, development of practical skills and motivational, emotional and attitudinal changes (Internal consistency: Cronbach-α ≥ .75) [21]. Apart from the adaptation to dentistry, the questionnaire was adopted largely unchanged. It consisted of 54 items and was subdivided into five main scales:

1. Social and communication competence, 2. Core competence training, 3. Acquisition of knowledge, 4. Teaching dentist and his/her patients, 5. Overall assessment. The answers were given according to a seven-level Likert rating scale (1 = ‘does not apply at all’ to 7 = ‘applies without limitation’). A school grade (1 = ‘very good’ to 6 = ‘failed’) was allocated for the assessment of the teaching dentist (Table 10).

#### Work shadowing reports (EVA III)

In addition, the students were requested to compose a freely-structured report, where they could describe and comment on the process of the work shadowing, as well as experiences and knowledge they gained.

#### Teaching practice evaluation questionnaires (EVA IV)

A questionnaire was also developed for the teaching dentists (see Tables 11 and 12, left column), which consisted of 16 items: 13 questions to be answered with ‘yes’ or ‘no’; school grades (1 = ‘very good’ to 6 = ‘failed’) were applied for rating three items.

### Analysis

The statistical analysis of the checklists and assessment questionnaires of competencies (EVA I), was done applying non-parametric procedures by means of the Friedman Test, including a post-hoc test, the Bonferroni-Holm correction (BiAS. for Windows, Version 11.06).

The evaluation questionnaires of students (EVA II) and teaching practices (EVA IV) were analysed descriptively (Microsoft Office Professional Plus 2010 Excel for Windows).

The unstructured free text reports (EVA III) were manually grouped according to their comments being more positive or negative, and suggestions for improvements from all were extracted.

## Results

### Response rate

The response rates are presented in Table 2.

**Table 2:** Response rates (students *n* = 61, teaching practices *n* = 27)

		n Response	Response [%]
EVA I	Checklists and assessment questionnaires of competencies	53	86.9
EVA II	Student evaluation questionnaires *n* = 54 items	54	88.5
EVA III	Work shadowing reports	51	83.6
EVA IV	Teaching practice evaluation questionnaires *n* = 16 items	48	78.7

The highest response rate was found in student evaluation questionnaire (EVA II) with an average of 88.5%.

### Individual results

#### Eva I

The individual results of the checklists and assessment questionnaires of competencies (EVA I) are shown in Tables 3 and 4.

**Table 3:** Results of EVA I: Overall evaluation of competencies

	Mean	SD	Mdn.	Min.	Max.
Self-assessment of students prior to work shadowing	2.93	0.52	2.95	1.67	4.54
Self-assessment of students post work shadowing	2.22	0.39	2.20	1.33	3.05
≙ Overall improvement of competencies	0.71*	0.43	0.67	0.00	2.23
External assessment by dentist after work shadowing	1.89	0.47	1.79	1.00	3.14
∆ Assessment (self-assessment – external assessment)	0.33*	0.36	0.29	−0.25	1.46

1 = ‘excellent‘, 2 = ‘good‘, 3 = ‘satisfactory’, 4 = ‘sufficient’, 5 = ‘insufficient‘, 6 = ‘failed’, ≙ = equals, ∆ = difference, *p < .05

**Table 4:** Results of EVA I: Main focusses and assessment of individual competencies

No.	Competency	A	B	C	D	E		F	
	CORE COMPETENCIES	[%]	Mean	Mean	Mean	Mean	SD	Mean	SD
1	Behaviour in the practice team	35.8	2.49	1.78	1.35	0.71*	0.61	0.43*	0.56
2	Behaviour towards patients	47.2	2.19	1.74	1.29	0.45*	0.56	0.46*	0.52
3	Dentist’s discussion techniques and anamnesis	35.8	2.93	1.99	1.75	0.94*	0.80	0.24*	0.56
4	Specialised knowledge in case reports	24.5	3.05	2.36	2.16	0.70*	0.57	0.20	0.52
5	Specialised knowledge in organisational matters	22.6	3.53	2.48	2.21	1.05*	0.61	0.27*	0.60
6	Specialised knowledge in accounting matters	26.4	4.64	3.47	3.21	1.17*	0.77	0.26	0.98
7	Specialised knowledge in personnel matters	13.2	3.96	3.06	2.60	0.90*	0.94	0.46*	0.96
8	Specialised knowledge in internal practice QM measures	18.9	4.27	3.46	2.88	0.81*	0.76	0.59*	0.80
9	Specialised knowledge in the patient admission process	15.1	2.95	2.30	2.05	0.64*	0.58	0.25*	0.46
10	Specialised knowledge in communication matters	17.0	2.75	2.14	1.80	0.61*	0.61	0.34*	0.71
11	Specialised knowledge in the management of emergencies	13.2	3.47	3.00	2.67	0.47*	0.70	0.33	0.77
12	Contents of the emergency kit	13.2	3.54	2.85	2.48	0.70*	0.82	0.38*	0.65
13	Awareness and acknowledgement of anamnesis and dental findings	34.0	2.50	1.97	1.73	0.53*	0.54	0.24*	0.47
14	Willingness to learn on own initiative and work independently	18.9	2.05	1.60	1.30	0.44*	0.71	0.29*	0.46
15	Composition of a case report	11.3	3.20	2.60	2.19	0.60*	0.66	0.39*	0.70
16	Behaviour in the communication with the dental laboratory	9.4	2.62	2.23	1.91	0.38*	0.50	0.32*	0.57
	ADDITIONAL COMPETENCIES								
1	Dental prophylaxis instruction	17.0	2.49	1.92	1.65	0.57*	0.57	0.26*	0.52
2	Scaling of calculus	15.1	2.53	1.69	1.58	0.83*	0.80	0.11	0.39
3	Restorative measures (non-adhesive)	9.4	2.57	2.21	1.97	0.36*	0.48	0.27	0.48
4	Restorative measures (adhesive)	30.2	2.48	1.87	1.77	0.61*	0.70	0.09	0.47
5	Restorative measures (indirect)	13.2	2.68	1.97	1.63	0.71*	0.73	0.34*	0.47
6	Endodontic measures	35.8	2.93	2.13	1.94	0.80*	0.71	0.19	0.59

1 = ‘excellent‘, 2 = ‘good‘, 3 = ‘satisfactory’, 4 = ‘sufficient’, 5 = ‘insufficient‘, 6 = ‘failed’, A = Frequency of the choice as main focus, B = Assessment of students prior to work shadowing, C = Assessment of students post work shadowing, D = Assessment by practicing dentist, E = Improvement (B-C), F = Difference between assessment by students - dentists (C-D), *p < .05.

On average, the students rated their knowledge in all assessed competencies prior to the work shadowing with the grade 2.93 ± 0.52 and after the work shadowing with 2.22 ± 0.39. Thus, the analysis showed a significant [*p < 0.05] overall improvement in the students’ self-assessed competencies by 0.71* ± 0.43 grades.

With an average of 0.33* ± 0.36, the dentists’ external assessment proved significantly higher than the self-assessment.

The greatest improvements were perceived by the students in the areas of accounting (1.17* ± 0.77), practice organisation (1.05* ± 0.61) and dentist’s discussions (0.94* ±0.80).

On average, each student had set 4.8 competencies as main focus with his/her dentist prior to the work shadowing. The most frequently chosen priority among students and dentists was the competency ‘Behaviour towards patients’ (47.2%). This was followed by the competencies ‘Behaviour in the practice team’, ‘Dentist’s discussion techniques and anamnesis’ and ‘Endodontic measures’, each with 35.85%.

#### EVA II

The individual results of the student evaluation questionnaires (EVA II) are shown in Tables 5, 6, 7, 8, 9 and 10.

**Table 5:** Results of EVA II in the scale ‘Social and communication competency’

No.	Item	Mean	SD
1	I have gained confidence in patient communication.	5.02	1.46
2	I was able to apply and improve my anamnesis techniques.	4.14	1.52
3	I experienced how important it is for chronically ill people to be in a familiar and social environment.	3.91	1.98
4	I was able to improve my competence in dealing with patients.	5.19	1.34
5	I feel more confident when dealing with the fears of my patients.	4.69	1.45
6	My tolerance for the social environment of patients has improved.	4.90	1.70
7	Occasionally, patients entrusted me with things that my work shadowing dentist did not yet know.	2.23	1.65
8	I take the costs of my orders into consideration.	4.83	1.62
9	I have learned to recognise the expectations of my patients.	5.04	1.37
10	I feel more confident in holding patient discussions after the work shadowing period.	4.79	1.76
11	I have learned to recognise the social problems of my patients.	4.23	1.67
12	Patients increasingly also told me personal things.	3.92	1.84
13	I learned to deal with fearful patients (e.g. children) in a patient manner.	4.75	1.50
	Total rating	4.43	0.79

1 = ‘does not apply at all’, 2 = ‘does mostly not apply’, 3 = ‘does rather not apply’, 4 = ‘partially applies’, 5 = ‘rather applies’, 6 = ‘mostly applies’, 7 = ‘applies without limitation’

**Table 6:** Results of EVA II in the scale ‘Core competence training’

No.	Item	Mean	SD
14	I was able to improve my competencies in dealing with the practice team.	5.26	1.46
15	I was able to improve my competencies in dealing with patients.	5.23	1.34
16	I was able to improve my competencies in holding dentist’s discussions and anamnesis.	4.72	1.36
17	I was able to improve my specialised knowledge on accounting matters.	3.93	1.91
18	I was able to improve my specialised knowledge on personnel matters.	3.91	1.63
19	I was able to improve my specialised knowledge with internal practice QM measures.	3.59	1.80
20	I was able to improve my specialised knowledge in ordering the admission of patients.	3.78	1.71
21	I was able to improve my specialised knowledge in communication matters.	4.58	1.47
22	I was able to improve my specialised knowledge on the management of emergencies.	3.47	2.05
23	I was able to improve my specialised knowledge on the content of the emergency kit.	3.50	2.15
24	I was able to improve my competence in learning on own initiative and working independently.	4.28	1.71
25	I was able to improve my specialised knowledge when composing case reports.	3.70	1.83
26	I was able to improve my behaviour in the communication with the dental laboratory.	4.30	2.01
	Total rating	4.17	0.62

1 = ‘does not apply at all’, 2 = ‘does mostly not apply’, 3 = ‘does rather not apply’, 4 = ‘partially applies’, 5 = ‘rather applies’, 6 = ‘mostly applies’, 7 = ‘applies without limitation’

**Table 7:** Results of EVA II in the scale ‘Acquisition of knowledge’

No.	Item	Mean	SD
27	After the work shadowing, I can order the problems of a patient according to their importance.	4.92	1.44
28	I can convey the content of a procedure to a patient clearly.	5.23	1.02
29	I now know how limit severe illnesses from bagatelle illnesses.	4.45	1.39
30	I read about medical disorders that were not familiar to me and increased my knowledge that way.	3.90	1.62
31	I received suggestions at the teaching practice on learning content that I can read about.	4.65	1.74
32	The patients I experienced motivated me to read about and acquire basic theoretical skills.	4.96	1.49
33	I can now recognise emergencies as such.	4.39	1.59
34	I learned about the implementation of the most important groups of medication at the work shadowing.	3.48	1.48
	Total rating	4.50	0.58

1 = ‘does not apply at all’, 2 = ‘does mostly not apply’, 3 = ‘does rather not apply’, 4 = ‘partially applies’, 5 = ‘rather applies’, 6 = ‘mostly applies’, 7 = ‘applies without limitation’

**Table 8:** Results of EVA II in the scale ‘My teaching dentist and his/her patients’

No.	Item	Mean	SD
35	I learned a lot from my teaching dentist on how to deal with patients.	5.98	1.28
36	I was able to learn from patients how they experienced and processed their condition.	4.66	1.51
37	I experienced a very individual approach to the conditions of the patients.	4.84	1.50
38	The interaction of my teaching dentist with the practice team was of role model quality to me.	5.87	1.53
39	I got to know patients with medical conditions of the most diverse dental areas.	4.59	1.82
40	I was able to discuss problems with the dentist regarding my personal career path.	5.02	1.66
41	The range of conditions and patients at my work shadowing practice were very divers.	4.66	1.82
42	My dentist has become a role model to me through his/her way of dealing with patients.	5.33	1.74
43	The patients showed me how they deal with their conditions.	3.98	1.51
44	I was also able to discuss my personal problems regarding my studies with my dentist.	4.64	1.86
45	The patients were very co-operative during the work shadowing period.	5.64	1.13
46	The patients took me seriously.	5.76	1.13
47	My teaching dentist demonstrated many examinations to me beforehand.	5.65	1.47
48	My teaching dentist regularly gave me feedback.	5.49	1.65
49	I found the feedback provided by my teaching dentist was motivating and useful.	5.76	1.41
	Total rating	5.19	0.60

1 = ‘does not apply at all’, 2 = ‘does mostly not apply’, 3 = ‘does rather not apply’, 4 = ‘partially applies’, 5 = ‘rather applies’, 6 = ‘mostly applies’, 7 = ‘applies without limitation’

**Table 9:** Results of EVA II in the scale ‘Overall assessment’

No.	Item	Mean	SD
50	I expanded my knowledge during the work shadowing.	5.59	1.41
51	I expanded my practical skills during the work shadowing.	3.82	2.03
52	I got to know the ethical aspects of the profession as dentist during the work shadowing.	4.94	1.72
53	On the whole, I am very satisfied with my teaching dentist.	6.33	1.23
	Total rating	5.17	1.06

1 = ‘does not apply at all’, 2 = ‘does mostly not apply’, 3 = ‘does rather not apply’, 4 = ‘partially applies’, 5 = ‘rather applies’, 6 = ‘mostly applies’, 7 = ‘applies without limitation’

**Table 10:** Results of EVA II Supplementary question

No.	Item	Mean	SD
54	School grade for the teaching dentist	1.58	0.93

1 = ‘excellent‘, 2 = ‘good‘, 3 = ‘satisfactory’, 4 = ‘sufficient’, 5 = ‘insufficient‘, 6 = ‘failed’

#### Scale ‘Social and communication competency’ (13 items)

The total rating of the scale ‘Social and communication competency’ was 4.43 ± 0.79.

On average, the students rated the following items the highest: ‘I was able to improve my competence in dealing with patients’ (5.19 ± 1.34), ‘I have learned to recognise the expectations of my patients’ (5.04 ± 1.37) and ‘I have gained confidence in patient communication’ (5.02 ± 1.46).

#### Scale ‘Core competence training’ (13 items)

The total rating of the scale ‘Core competence training’ was 4.17 ± 0.62.

On average, the students rated the following items the highest: ‘I was able to improve my competencies in dealing with the practice team’ (5.26 ± 1.46), ‘I was able to improve my competencies in dealing with patients’ (5.23 ± 1.34), and ‘I was able to improve my competencies in holding dentist’s discussions and anamnesis’ (4.72 ± 1.36).

#### Scale ‘Acquisition of knowledge’ (8 items)

The total rating of the scale ‘Acquisition of knowledge’ was 4.50 ± 0.58.

On average, the students rated the following items the highest: ‘I can convey the content of a procedure to a patient clearly’ (5.23 ± 1.02), ‘The patients I experienced motivated me to read about and acquire basic theoretical skills’ (4.96 ± 1.49) and ‘After the work shadowing, I can order the problems of a patient according to their importance’ (4.92 ± 1.44).

#### Scale ‘My teaching dentist and his/her patients’ (15 items)

The total rating of the scale ‘My teaching dentist and his/her patients’ was 5.19 ± 0.60.

On average, the students rated the following items the highest: ‘I learned a lot from my teaching dentist on how to deal with patients’ (5.98 ± 1.28), ‘The interaction of my teaching dentist with the practice team was of role model quality to me’ (5.87 ± 1.53) and ‘I found the feedback provided by my teaching dentist was motivating and useful’ (5.76 ± 1.41).

#### Scale ‘overall assessment’ (4 items)

The total rating of the scale ‘Overall assessment’ was 5.17 ± 1.06.

On average, the students rated the following items the highest: ‘On the whole, I am very satisfied with my teaching dentist’ (6.33 ± 1.23) and ‘I expanded my knowledge during the work shadowing’ (5.59 ± 1.41).

#### Supplementary question

The school grade for the teaching dentist by the students was on average 1.58 ± 0.9.

#### EVA III

The student’s subjective free text reports on work shadowing described the course of the work shadowing and the treatment measures that were observed. Here, they had the opportunity to provide feedback on their work shadowing experience, either in the form of praise or criticism, including suggestions for improvement and an assessment of the dentists’ teaching engagement.

Table 11 represents excerpts from the positive and negative feedback, as well as improvement suggestions.

**Table 11:** Results of EVA III: Excerpts from the work shadowing reports

Positive feedback	
„I have gained a lot of knowledge, important experience, immense value, tips and tricks, as well as a lot of joy for having chosen the right course of study. My curiosity and passion for dentistry has grown and I look forward to taking on the coming challenges that this profession entails.”; “After this week’s work shadowing with Dr. Schmidt [*name changed*], my enthusiasm for dentistry has grown significantly! On a final note, I must emphasise how educational and sensible this work shadowing has been for my future career path as a dentist.”; “I will remember this work shadowing week for a long time. Despite the brief time period, the knowledge I have gained is immense – both on a human and specialised level.”; “Besides, it was extremely comforting to receive 1-to-1 attention that cannot be achieved at the university, due to the large number of students. On the whole, I assess the project as being a positive experience and a very important addition to my degree course. I am ending this enlightening week in the hope of leaving the university after my exams to enter my working life that little more experienced.”; “I learned a lot in these two days and would most of all liked to have completed the work shadowing in two weeks rather than two days, as the gain in knowledge is enormous. Thank you for this great opportunity […]!; “Finally, I can only say that my time during the work shadowing internship was enriching to me and I would not like to have missed out on the experiences.”; “an interesting view outside of the box”; “My work shadowing […] was really amazing. […] I was thrilled by both the practice and assistants, as well as the dentists.”; “The work shadowing at the dentistry practice Dr. med. Dent Fischer [*name changed*] was extremely informative and encouraging. It was motivating and above all made me more confident in conversing with patients.”; “The learning effect is extremely sophisticated and the work shadowing time is definitely well-invested. I am sure one can gain a wide range of knowledge from it at a later stage.”; “I expanded my knowledge during the work shadowing days and would do it again anytime, to improve my knowledge.”; “I was very well-treated and felt extremely welcome. […]The entire practice team became a role model to me and I would be glad to work shadow again.”	
Negative feedback	
“Unfortunately, the work shadowing period was much too short to gain a clear view over the full range of duties”; “Unfortunately, my dental skills only slightly improved. On the whole, I was not allowed to do a lot independently and had the feeling my doctor was also not very interested.”; “However, in my opinion, the work shadowing day is not enough to convey a suitable overview of the daily life of a practice.”; “I find that participating in a project at this point in the degree is too late […].”; “The work shadowing was not good. From Dr. Weber [*name changed*] I could only really gather how things should not be done.”; “Unfortunately, in retrospect, the duration of the work shadowing was far too short.”; “Due to a lack of time, it was not possible to address topics such as accounting, for example, in which we have not been trained in any way up to now.”; “[…], however, it is not possible to become fully acquainted with a practice in such a brief period of time.”; “I found that the internship was too early after the 6th semester.”	
Suggestions for improvement	
“As a suggestion for improvement, I would like to add that it would be far more interesting for students if they could involve themselves in the treatment more, through assisting or similar […].”; “[…] something like this should be on a voluntary basis.”; “It would be better to offer a seminar in bookkeeping at the university clinic or something similar.”	

The qualitative evaluation of the collected reports is planned to be the subject of future investigations.

#### EVA IV

The evaluation results from the teaching practices are shown in Tables 12 and 13.

**Table 12:** Results of EVA IV: ‘Yes/no’ questions (*n* = 48 practice evaluations)

No.	Item	Yes [%]
1	Were you satisfied with the work shadower?	100
2	Was the work shadower reliable?	100
3	Was the work shadower punctual?	100
4	Was the work shadower well-prepared?	97.9
6	Did you hold specialised conversations with the work shadower?	100
7	Did you find there was sufficient time to pay attention to the work shadower?	33.3
8	In your opinion, did the competencies of the work shadower on the following topic improved during the period at your practice?	
	A: Accounting	37.5
	B: Management of emergencies	27.1
	C: Contents of the emergency kit	25.0
	D: QM measures	29.2
	E: Organisation	75.0
	F: Dentist’s discussion techniques and anamnesis	87.5
	G: Behaviour towards patients	89.6
	H: Prophylaxis instructions	75.0
9	In your opinion, did the work shadower achieve his/her goal?	95.8
10	Were you satisfied with the project management?	87.5
11	Were you well-informed on the project?	89.6
12	Did the correspondence work well?	89.6
13	Was the foreseen period of 4.5 days enough to gain a view into the daily life of a practice?	37.5
14	Do you have improvement suggestions/requests for the project management?	43.8

**Table 13:** Results of EVA IV: School grades

No.	Item	Mean	SD
5	I give the work shadower the following school grade:	1.27	0.50
15	I give the project leaders the following school grade:	1.91	0.80
16	I give the project the following school grade:	1.69	0.89

1 = ‘excellent‘, 2 = ‘good‘, 3 = ‘satisfactory’, 4 = ‘sufficient’, 5 = ‘insufficient‘, 6 = ‘failed’

They show a mean grading of the work shadowers with 1.27 ± 0.50 and a 100% satisfaction, but only 37.5% of the practices think that the time was enough to gain a view into the daily life of a practice.

## Discussion

### Assessment by students

In the area of medicine, numerous studies support the advantages of the individual supervision of students in teaching practices [20, 21, 23]. A meta-analysis published in 2011 on the impact of outside training experiences at medical universities, showed that students experience a positive learning influence that they value greatly [11]. In the area of dentistry, many studies can be found addressing the value of outreach experiences [33–43], whereas far less exist on work shadowing or internships within a general dental practice. The study group of Craddock could, however, show that students of dentistry also value a traineeship in practices highly and would recommend it further [32]. The overall assessment by students portrayed in the present study reflects a similar result: the students express a growth in knowledge during their work shadowing experience and demonstrate great satisfaction in regard to the teaching dentists. In the evaluation of the scale ‘Core competence training’, it is clear that students perceived an improvement particularly in regard to conversation techniques, taking case histories and appropriate behaviour when dealing with the practice team and patients. In this way, parts of the NKLZ learning goals of the communicator were illustrated that indicate that communication skills are of central importance in the profession of dentistry [48]. This is also supported by Johnston and Boohan, who, after comparing the medical and dental courses of practices and teaching hospitals, concluded that fundamental clinical abilities were taught best in a practical surrounding [52]. In addition, the student evaluation showed that practicing dentists acted as role models in regard to the behaviour of both patients and the practice team. This fact should certainly be regarded as a great gain, since the discovery of a role model encourages medical thought-processes and behaviour [53], as well as facilitates the consideration of medical ethics, even if not consciously taught [54].

Assessments of competence expansion of practical skills received low evaluation values, together with the knowledge of personal patient information. Since the study was primarily concerned with work shadowing, without any specific active treatment or development of conversation techniques, these results are not very surprising. It is also equally clear that it was very difficult to address medication, general medical cases and the management of medical emergencies sufficiently during the course of the work shadowing. These general medical topics were obviously forced into the background by routine dental issues. In the future, it could be possible to address the importance of these topics individually, through pre-designated train-the-teacher sessions.

### Assessment by practicing dentists

The willingness of the practices to participate in the study was significantly high and a positive overall assessment clearly demonstrated that the practitioners very much welcome such a form of teaching. Equally high satisfaction was demonstrated towards the work shadowers by the dentists. Using previous studies with projects in medical teaching practices, there are many possible reasons for this: besides improved self-esteem and the gratification of teaching [27, 28], one can see that the presence of a student has a positive effect on the profile and team spirit of a practice [30]. In 2012, May et al. identified further motives for becoming a teaching practice. Here, the main motivations proved to include working as a medical teacher, the improvement of medical teaching, passing on knowledge and helping others [29]. This motivation to act as a teacher for both patients and relatives, as well as students and employees, is also conveyed to future dentists via the NKLZ role model of the scholar [48]. In this way, this evaluation suggests that the described results in medicine can be applied to teaching in dental practices. This is supported by the Craddocks results that show that a student internship enriches the everyday life of a practicing dentist and is categorised as being worthwhile and recommendable [32].

Parallel to the evaluation by students, the practices also suggest that there were competencies that were less focussed on during the course of the work shadowing. Here also, the low result values of emergency topics became apparent. All-in-all, approximately two-thirds of the practicing dentists considered the work shadowing time period insufficient for gaining insight into the everyday life of a practice. The reason for this lies largely in the voluntary nature of the study. Due to the lack of curricular correlation, only a mere offer of a five-day work shadowing time period was possible. The extent to which advantage was taken of a full five-day period, lay in the hands of the students themselves. This ranged widely from only one work shadowing day, to students, who extended the practical duration by their own initiative to ten days. For this reason, one cannot deduce from the results how long work shadowing should be, although one can interpret the clear expression of the retrospective wish for more time as an indication of the high acceptance of the venture and the probable value of an extension.

### Acquisition of competencies

One can deduce what students expected to gain from the practice period, by the choices of main focusses they provided prior to their work shadowing (Table 4, column A). When one considers that dentistry is a patient-oriented course of study in Germany, it is rather surprising that the overwhelming majority selected their main focus as being ‘behaviour around patients’. One can attempt to explain this by taking the student population into consideration: as it largely consists of Phantom Course graduates of Operative Dentistry (6th study semester), this dealt with students who were on the verge of treating their own first patients. From the observation of an experienced operator, the work shadowers obviously hoped primarily to gain basic skills in communication, in preparation for their coming treatment courses. The high interest in anamnesis and clinical dental findings also appears to be founded on this fact.

On average, the students acknowledged a significant improvement in all areas of competency through the work shadowing that they graded in their self-assessment. It is apparent that the main differences in the self-evaluation are in areas unrelated to the most frequently-chosen main focusses. This implies that there is little correlation between the pre-existing interests and the perceived gain in competencies. The work shadowers perceived their greatest improvement in the areas of accounting, organisation, personnel matters and dentistry-related communication – all being areas that are barely or not at all covered in university courses. Only after these areas, do perceived improvements in the specialised fields become relevant. This allows us to deduce that through individual attention and a specific teaching environment, especially new subject matter can be introduced in a relatively short period of time. When the assessments of the evaluation questionnaires (EVA I) are examined, however, one can see that despite a significant improvement by approximately one school grade in the competence comparison, precisely these practice-specific areas were assessed rather inconclusively. A possible explanation for this could be that despite a significant increase, the work shadowers categorised the potential gain of knowledge in these new areas initially so high that it was not possible to confirm without a limitation of the improvement achieved.

On the whole, the triangulation across the assessment of practicing dentists shows a significantly better external assessment in comparison to the self-assessment by students. In the individual comparison of competencies, however, one can see that this significance is only present in approximately two-thirds of the areas. Particularly in the additional competencies of the treatment measures, no great differences can be recognised between the two forms of assessments, which is probably due to the limited evaluation ability in the scope of a work shadowing period without proper interaction with patients.

According to the current state of research, there are no studies that investigate the acquisition of competencies during work shadowing at general dental practices by means of the self-assessment of students over a given period of time, as this study does. For comparison, however, the self-assessments of dentistry outreach periods can be used. Smith analysed the outreach experiences of British dental students between 2006 and 2011 in a variety of studies [33, 41–43]. These include competence comparisons of self-evaluations, above all relating to self-confidence in clinical situations, anamnesis and planning of treatments. Similar to the results of this study, all of these competencies were estimated to improve through external teaching experiences [41, 42]. Especially with regard to the literature about workplace learning initially mentioned, we can conclude that these results can be considered for teaching in dental practices: Our results show that dental students are also able to enhance skills in a practical environment and that the dentist as an experienced person is able to convey information and requirements for new areas in a very elementary way [6, 8]. It was possible to combine dental theory with clinical practice [9], and also the competence improvement shown in communication and case reports corresponds to the results from studies about workplace learning [12, 13]. Through the simultaneous consideration that the competencies investigated here are all illustrated in the NKLZ, our results allow us to assume that the work shadowing periods carried out represent a sensible addition to academic dental teaching at a university [48].

### Reports

The freely-structured reports illustrate a largely positive and partially even very enthusiastic viewpoint of the students. The majority of work shadowers categorised the experiences they had as interesting and worthwhile. They recognised it to being a sensible addition to their studies and a good opportunity for gaining insight into the daily life of a practice. In most cases expressing negativity, this was related to the fact that the work shadowing was perceived as too short. A few reports indicate that the teaching motivation differed depending on the practice. In 2016, a model requirement profile for co-operative practices comparable to that in general medicine, was created in Germany for the future establishment of dental teaching practices [25, 45, 55]. The implementation of this profile in combination with train-the-teacher sessions, may ensure the motivation for dental teaching.

### Work shadowing vs. internship

During the conception of this study, it was initially limited to a work shadowing period, i.e. the observation and attendance of treatment situations. The independent treatment of patients in the teaching practice or assisting of students surrounding the scope of an internship, were first considered but not initially incorporated, for insurance-related and organisational reasons. Even the new Dental Licensure Act regards the clinical traineeship as an insight into the career path without active responsibility for patients [3]. However, the current student votes support studies, that demonstrate a clear willingness to take over an active role on part of students, within the scope of teaching outside the university [19, 37]. Furthermore, examinations of the productivity of students being able to gather double to three times the amount of treatment experience at external treatment institutes, in comparison to a similar time period at a university [32, 34, 43], show that this results in a steeper learning curve and is a great advantage in their career development [39].

In Germany, the foundation for an active dental internship was laid in Saxony in 2016: the University of Dresden succeeded in cooperating with the Saxon Chamber of Dentists and an insurance company, to establish co-operative practices and facilitate treatment by students that was legally liable [45]. An expansion to other German universities would certainly be a significantly positive development.

### Train-the-teacher

In the present study, mostly dentists without a background in education or teaching experience functioned as mediators of theoretical and practical knowledge. The results and the high level of satisfaction on both sides show that this transmission was nevertheless successful. However, a search of the literature reveals that the most important basis for workplace learning should be professionally trained and well-prepared teachers [56]. As already implied, it would be beneficial to train the participating dentists in some basic educational skills through separate courses to improve the student’s learning experience. Such train-the-teacher sessions prior to a student internship at practices have many advantages:

A precise organisational planning and cooperation is facilitated right from the start, a platform for encouraging learning motivation is offered and problems that could arise on part of the practice with regard to time management and costs could be addressed [30]. In addition, a train-the-teacher session is fundamentally important for choosing main areas, comparing content and unifying specialties [32, 35]. Furthermore, other investigations show that lecturer training is essential for gaining an easy entry into the role as ‘scholar’, that it influences the later teaching duty effectively and in that way also facilitates the formation of a teaching community [30, 57, 58].

Unfortunately, such a training session could not be implemented in this study, for reasons of financial funding. The authors, however, regard such training as indispensable, in order to establish academic learning practices in a study of dentistry.

### Limitations

One limitation in the investigation of the acquired competencies certainly lies in the fact that self-assessment was chosen as the tool of data collection. In this way, several subjective influences affect the results. On one hand, the Hawthorne Effect, since both the initial and concluding grades, as well as the external assessment took place within the same checklist. One could conceive that the students and dentists estimated achieving greater improvement than may have normally been acknowledged otherwise, due to the awareness of participating in a study and having knowledge of the initial grades. On the other hand, it is generally hard to differentiate whether these incorporate only perceived or actually improved competencies. The final assessment by the students appears to be objectively confirmed by the dentists or even partially evaluated significantly higher. However, for a more precise assessment as to the applicability of this effect on the improvement of competencies, a more objective tool for the acquisition would have to have been chosen. One possibility for this might be found in Smith et al., who by means of scenarios with actors, assessed the conversation and planning competencies of students before and after an outreach internship, through external assessors [41]. Similar scenarios could also be applied to follow-up studies on the influence of dental teaching practices in some of the indicated areas of competency.

An evaluation of the patient’s opinion during the intervention was not possible for organisational reasons. There is evidence, however, that the treatment provided in the scope of a teaching practice is also advantageous to patients: Grant and Robling indicated that patients are thankful for being able to follow a discussion on their treatment and feel more involved in their care that way [27]. Also in the outreach area, child patients and their parents proved to be highly satisfied with the student-based treatment [36, 38].

For follow-up studies, the incorporation of the view of patients would definitely offer an enrichment to the whole teaching community from university to practices.

## Conclusions

Taking into account the mentioned limitations, the following conclusions could be drawn from the present study, with regard to the initially posed questions:

- Both on the part of the students, as well as the practicing dentists, a high degree of acceptance and satisfaction is shown in regard to the work shadowing.
- The students see the dentists as motivating role models, and acknowledge a growth in knowledge and an improvement in their behaviour when dealing with patients and the practice team.
- The students acknowledge a significant improvement in their specialised, communication and social competencies. The greatest improvement was perceived to occur in the areas of accounting and practice organisation, as well as dentist’s discussion techniques. In scope of this, the assessment was confirmed by the dentists’ assessment and partially evaluated significantly higher.
- Work shadowing in teaching practices presents a sensible addition to academic teaching at a university, since the students estimate that a further range of competencies can be covered.
- Both the students and practicing dentists would like the duration of the work shadowing to be longer.
- The students would like to play a more active role.

The goal of further investigations should include substantiation of the described subjective results with more robust objective measures.

## Abbreviations

BZÄK: Bundeszahnärztekammer (German Dental Association)
EVA: Evaluation
NKLZ: Nationaler Kompetenzbasierter Lernzielkatalog Zahnmedizin (National competence-based learning goal catalogue for dentistry)
